# Treated *Rhizophora mucronata* tannin as a corrosion inhibitor in chloride solution

**DOI:** 10.1371/journal.pone.0200595

**Published:** 2018-08-08

**Authors:** Augustine Agi, Radzuan Junin, Muhammad Rasol, Afeez Gbadamosi, Radhika Gunaji

**Affiliations:** 1 Department of Petroleum Engineering, Faculty of Chemical and Energy Engineering, Universiti Teknologi Malaysia, UTM Skudai, Johor Bahru, Malaysia; 2 Department of Petroleum Engineering, University of Port Harcourt, Port Harcourt, Nigeria; 3 Department of Chemical Engineering, Ladoke Akintola University of Technology, Ogbomoso, Nigeria; Cranfield University, UNITED KINGDOM

## Abstract

Treated *Rhizopora mucronata* tannin (RMT) as a corrosion inhibitor for carbon steel and copper in oil and gas facilities was investigated. Corrosion rate of carbon-steel and copper in 3wt% NaCl solution by RMT was studied using chemical (weight loss method) and spectroscopic (FTIR) techniques at various temperatures in the ranges of 26–90°C. The weight loss data was compared to the electrochemical by the application of Faraday’s law for the conversion of corrosion rate data from one system to another. The inhibitive efficiency of RMT was compared with commercial inhibitor sodium benzotriazole (BTA-S). The best concentration of RMT was 20% (w/v), increase in concentration of RMT decreased the corrosion rate and increased the inhibitive efficiency. Increase in temperature increased the corrosion rate and decreased the inhibitive efficiency but, the rate of corrosion was mild with RMT. The FTIR result shows the presence of hydroxyl group, aromatic group, esters and the substituted benzene group indicating the purity of the tannin. The trend of RMT was similar to that of BTA-S, but its inhibitive efficiency for carbon-steel was poor (6%) compared to RMT (59%). BTA-S was efficient for copper (76%) compared to RMT (74%) at 40% (w/v) and 20% (w/v) concentration respectively. RMT was efficient even at low concentration therefore, the use of RMT as a cost effective and environmentally friendly corrosion inhibiting agent for carbon steel and copper is herein proposed.

## Introduction

The oil and gas industry use a lot of metals in their daily activities, depending on the application. Carbon steels are the most widely used material for downhole tabular, oil tubing, flow lines, transmission pipelines, casing, this is because of their low cost [1, 2]. Surface equipment such as electrical power lines, pipelines for industrial utilities including sea water, heat conductors and exchangers are made of copper alloys. This is because of its high electrical, thermal conductivity, mechanical strength and its unique properties.

Be that as it may, these two metals irrespective of their unique properties are very susceptible to corrosion. Copper is very sensitive to chloride ions, little amount of Cl^-^ can form an unstable film, CuCl, soluble chloride complexes leading to rapid corrosion rate in sea water and chloride environment. It is well accepted that anode dissolution of metals in chloride environment is influenced by chloride ions concentration, at a concentration lower than 1 M, the dissolution of most metals occur which can no longer protect the metal from reacting with chlorides [3]. Corrosion problem therefore, represent a large portion of the total cost for the oil and gas industry every year worldwide.

Corrosion can occur in galvanic form, which is associated with the use of different dissimilar materials or as a result of two metals in contact with each other in gaps between two surfaces to cause crevice corrosion. Also, fretting corrosion can occur at the boundary of two contacting surfaces which oscillate relative to each other when machine or equipment vibrate, while the electro-corrosion is under the influence of external source of constant current or interrupted undirected current [4]. It is therefore, almost impossible to prevent corrosion but it is possible to control it. Appropriate corrosion control can help avoid many potential problems which result to loss of life, negative social issues, water resources and environmental pollution. To prevent most metals from corrosion, the use of organic compounds as corrosion inhibitors are employed.

These organic compounds are found mostly in plant extract, which can be extracted at low cost. Naturally occurring substance such as tomatoes peels [5]; *sida acuta* [6]; *Elaeis guineesis* frond lignin, Tinospora extract [7,8]; *Griffonia simplicifolia* extract [1], *Melia azedarach* [9], Areca nut [10] have been used as corrosion inhibitors at different environmental conditions, including various tannin extract such as chestnut tannin [11]; *Rhizophora apiculata* tannin [2, 12, 13, 14, 15]; Black wattle tannin [16].

**Fig 1:**
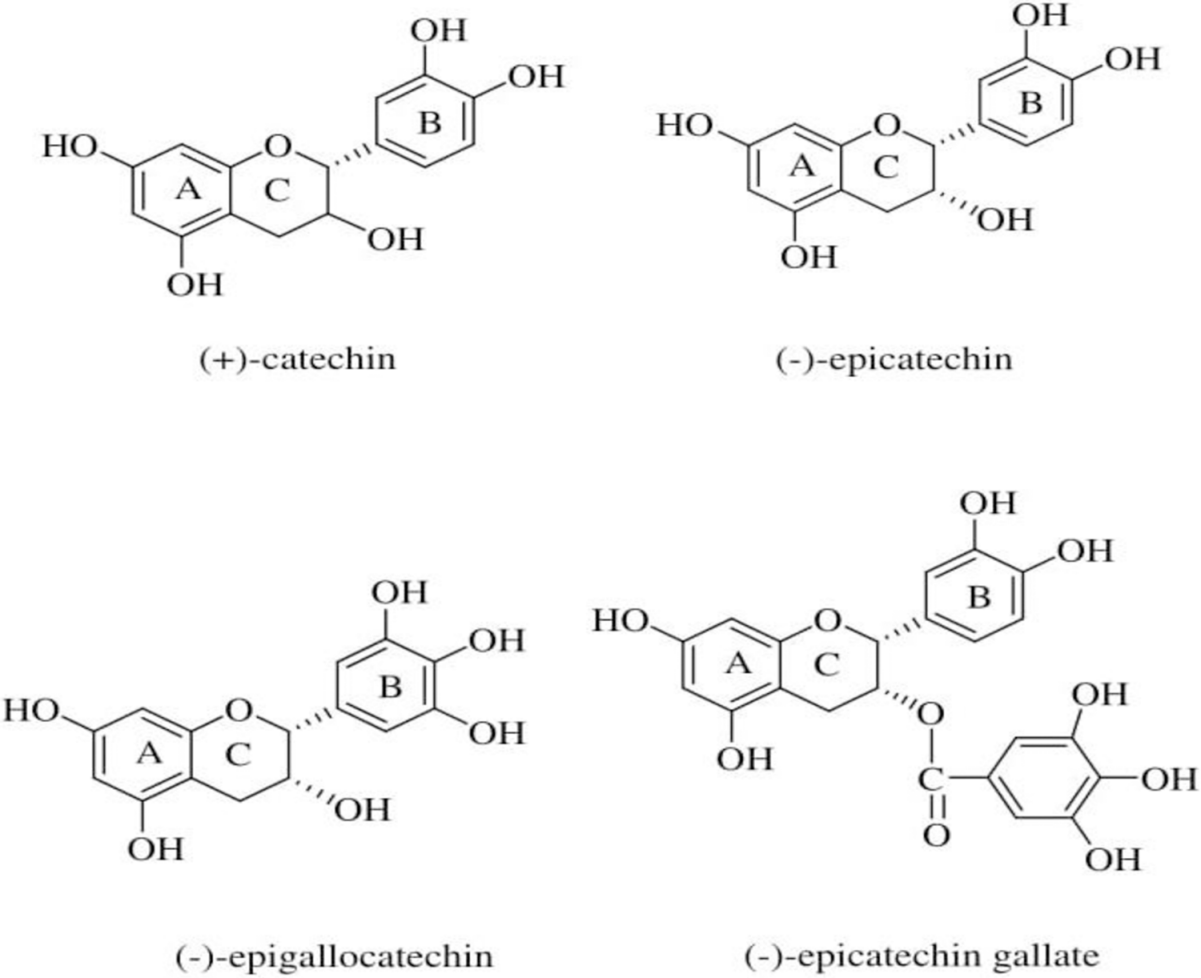
Chemical structure of flavonoid monomers of RMT [17].

Tannin has two classes of polyphenolic compounds, the hydrolysable and condensed tannin. The hydrolysable tannin is mostly from fruits, pods while the condensed tannin are mostly found in wood barks. Tannin consist of four flavonoid monomers such as catechin, epicatechin, epigallocatechin and epicatechin gallate [17]. The chemical structure of the flavonoid monomers is shown in Fig 1.

These monomers can adsorb ions or molecules on the metals surface to form a protective layer on the metal. The OH^-^ group on the aromatic rings can form chelates with iron and other metals such as copper [18, 19]. Previous studies on tannin concentrated on *Rhizophora apiculata* and mimosa in acidic solution were evaluated on mostly steel [2, 11, 12, 13, 14, 15, 16, 20]. Whereas, studies on copper and its alloy in NaCl solution used the commercial benzotriazole as the inhibitor [21, 22, 23, 24, 25].

Generally, the most important criteria for the formulation of corrosion inhibitor for field application is the stability and performance of the inhibitor [26, 27]. Before it is considered for field application, suitable laboratory tests are performed for a particular application to evaluate its suitability [27, 28, 29]. Presently, weight-loss measurement is the most accurate, reliable and precise method for determining general and pitting metal corrosion rate of oil and gas pipeline [26, 30]. This is because the experimentation is easy to replicate, even though it requires long exposure time, the simple procedure reduces the tendency to introduce systematic errors. Majority of oilfield testing procedures for evaluation of corrosion inhibitors are done using this method compared to the faster electrochemical technique [26]. Although, the electrochemical technique is a non-destructive, quantitative technique, but the difficulties in studying complex corrosion system as the marine environment and rust/metal complicates the electrochemical corrosion process [30]. The rust redox reaction, mass transportation through the rust, microorganism propagation in porous rust, electric charges movement between interface, makes corrosion rate between electrochemical and weight-loss to differ [31].

Papavinasam et al. [29] compared the corrosion technique for monitoring corrosion inhibitors in oil and gas pipelines. They reported that the polarisation resistance (Rp) measurement is less reproducible and uncertain compared to the weight-loss. Since the value of the anodic and cathodic Tafel slopes are needed in determining the corrosion rate. The electrochemical impedance spectroscopy (EIS) technique was less recommended due to poor reproducibility, development of a physical model and long measurement time. Although, the electrochemical noise was recommended, no satisfactory method has been developed for the presentation and interpretation of the results. The hydrogen permeation measurement on the other hand does not correlate with real corrosion in oil and gas pipeline. Also, the Tafel extrapolation method for the determination of corrosion density from which corrosion rate is calculated causes problems. Oxide formation, multiple anodic and cathodic reaction occurring simultaneously, concentration polarization causes the deviation from the original Tafel theory leading to corrosion error. It is therefore, not recommended to calculate inhibition efficiency and corrosion rate from Tafel plot measurement [26, 29]. Electrochemical technique is useful in obtaining other information such as the thermodynamics of the redox process, the kinetics of the electron transfer, presentation of the properties of the surface structure and corrosion phenomena [26, 29]. Weight-loss measurement followed by characterisation of the pits are by far the most reliable technique for monitoring the effect of corrosion inhibitors on uniform and pitting corrosion rate in the oil and gas industry [26, 29].

Exploitation of oil and gas has gone into deep pay zones in the subsurface, understanding the effect of temperature on corrosion inhibitor is important. Since temperature at the surface is different from bottom-hole temperature. The geothermal gradient is the difference in temperature per unit well length as on goes down the well. It is the increase in temperature with depth in the earth’s interior. The universal geothermal gradient is about 40°C/km, an average 25°C/km of depth (1°F per 70ft of depth) [1, 32].

Therefore, in this study treated *Rhizophora mucronata* tannin (RMT) inhibition on carbon-steel and copper in chloride solution is investigated and compared with commercial inhibitor sodium benzotriazole (BTA-S). The corrosion rate data from the weight-loss method was compared with the electrochemical method. The best RMT concentration was determined and used to evaluate the effect of temperature on the corrosion rate and inhibitive efficiency of the tannin.

## 2.0 Materials and methods

### 2.1 Materials

Mangrove bark sample (*Rhizophora mucronata*) was obtained from Tanjong Langsat mangrove forest Johor, Malaysia. No specific permission was required for obtaining the mangrove bark from Tanjong Langsat mangrove forest. This is because the forest is not under conservation. Rhizophora mucronata is not an endangered species, it is used by the locals to produce charcoal. And the bark is treated as a waste, and can be found littering the whole area. Carbon steel of size 50.4mm x 2.54mm x 6mm and copper 50.4mm x 2.54mm x 3mm were used for the corrosion test. The carbon steel and copper were supplied by AMS Light Metal Sdn. Bhd. Johor, Malaysia. NaCl was used as the electrolyte; it was supplied by Acros Organic Company with a purity of 99%. The commercial inhibitor BTA-S was purchased from Parchem, NY-USA.

### 2.2 Tannin extraction

The extraction of the tannin was carried out by total immersion of the ground bark and heated in 3L of distilled water as described elsewhere [27].

### 2.3 Infrared spectroscopy test

The spectrophotometry was carried out in a direct transmittance mode using the Perkins-Elmer 180 spectrometer. The region between 500–4000 cm^-1^ of wavelength was applied. The sample was prepared according to the potassium bromide (KBr) technique at a mass ratio of 1:100. The mixture was compacted for 10 minutes using a compactor to produce a flat transparent plate. The transparent plate was tested, and the interpretation of IR spectra was done using the Perkin Elmer Software.

### 2.4 Corrosion inhibition studies

**Fig 2:**
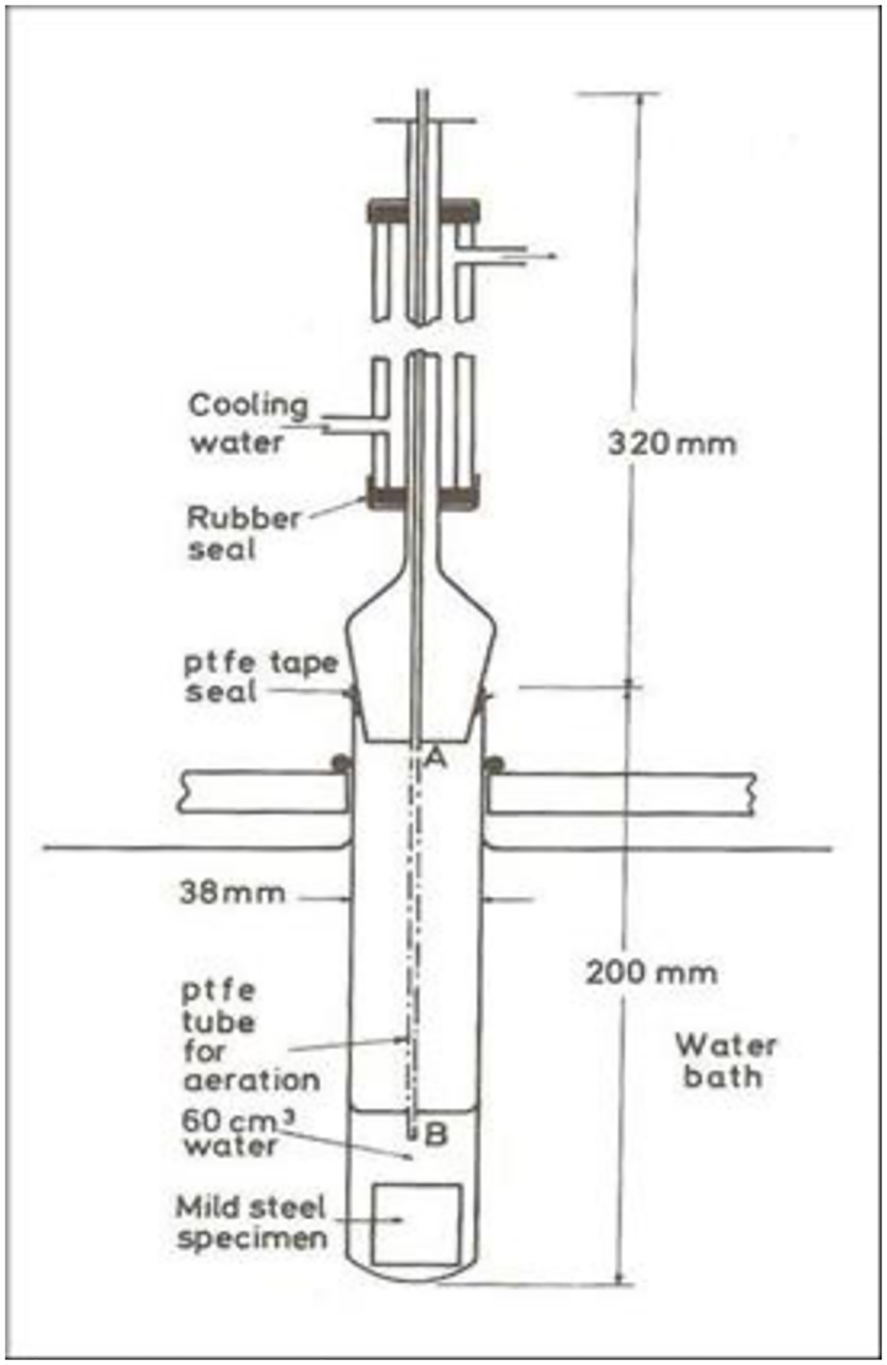
Schematic of the apparatus used for studying the effect of temperature on corrosion of metal.

The standard test method (ASTM STP 1000 [33]) was used for the experiment. Six apparatus labelled A-F were used, the schematic for the apparatus is shown in Fig 2. The treated tannin concentration at room temperature was varied (5, 10, 20, 30, and 50% w/v) to determine the best concentration. The effect of temperature on corrosion rate was studied at 26°, 50°, and 90°C. In this study, 40% (w/v) concentration of commercial inhibitor BTA-S was used. The experiment was conducted under total immersion of specimen. Each specimen of carbon steel and copper was placed separately in the apparatus for two weeks, after which it was removed cleaned and weighted. The corrosion rate (CR) was calculated using the formula:

$$CR=\frac{22300\;\Delta W}{ADT}$$ (1)

Whereas, Δ*W* is the weight loss (g), A is the surface area (cm^2^), D is the density of metal (g/cm^3^) and T is the time of exposure (days).

And the inhibition efficiency (IE) was calculated using the formula;

$$IE=1-\frac{w_{i}}{w_{o}}\times 100$$ (2)

Whereas, w_o_ is the weight loss without inhibitor, w_i_ is the weight loss with inhibitor.

The electrochemical data were determined analytically. The weight-loss parameters were converted to electrochemical by applying the Faraday’s law:

$$i_{corr}=\frac{nF\Delta m}{MtA}$$ (3)

whereas, i_corr_ is current density (A/cm^2^) which stands for corrosion rate, Δm is weight loss due to corrosion (g), n is the valence, F is the Faradays constant (96,500 coulombs), M is the molecular weight of the metal (g/mol), t is time (days), A is the area of metallic surface exposed to corrosion in cm^2^.

## 3.0 Results and discussion

### 3.1 Infrared spectroscopy

#### (a) RMT

**Fig 3:**
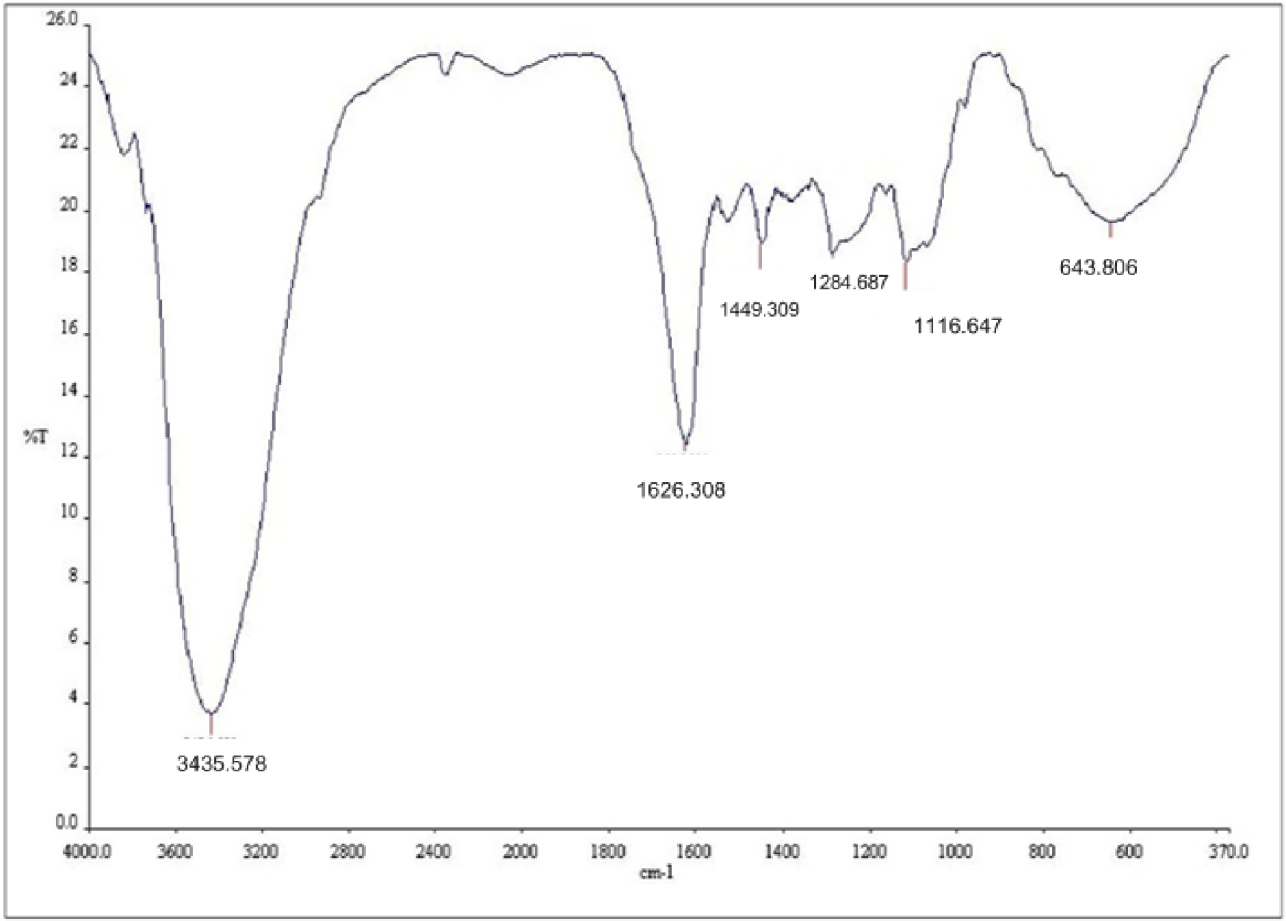
FTIR spectra for RMT.

The result of FTIR for RMT is shown in Fig 3. The reflection shows a 25% transmittance. The absorption has a broad spectra band between 3700–2700 cm^-1^ with the maximum absorbance at 3435.578 cm^-1^ which shows the presence of the hydroxyl group [3, 12, 16]. The reduced intensity from 25%–3.5% (transmittance y-axis) of the broad peak at 3435.578 cm^-1^ shows a reduction of the free OH group [16, 34]. The broad OH group shows the presence of phenols and sugar which is contained in catechin [15]. Catechin is the main constituent of condensed tannin which acts as an anti-oxidant to inhibit oxidation of molecules present in metals. The band from 2973–1700 cm^-1^ was due to the C-H stretching vibration assigned to the methyl and methylene groups [35]. The wavenumber of 1626.308 cm^-1^ corresponds to the presence of aromatic group. The C-C stretching attributed to CR_2_-CHR-CR(SO_3_^2-^) structure caused by the opening of the pyran ring during sulfitation of flavonoid tannins [35]. Other peaks occurring at 1449.709, 1284.687 and 1116.647 cm^-1^ was assigned to the ethereal C-O stretching vibration arising from the pyran-derived ring structure of the tannin [12, 36]. The wavenumber at 1284.687 cm^-1^ shows presence of flavan-3-ols and procyanidins. The peak at 1116.647 cm^-1^ can also be observed in gallic acid and tannic acid (Gallo tannin), which is as a result of C-O stretching and O-H deformation [36]. Smaller peak at 643.806 cm^-1^ corresponds to the substituted benzene group [3, 12, 16].

#### (b) Commercial inhibitor BTA-S

**Fig 4:**
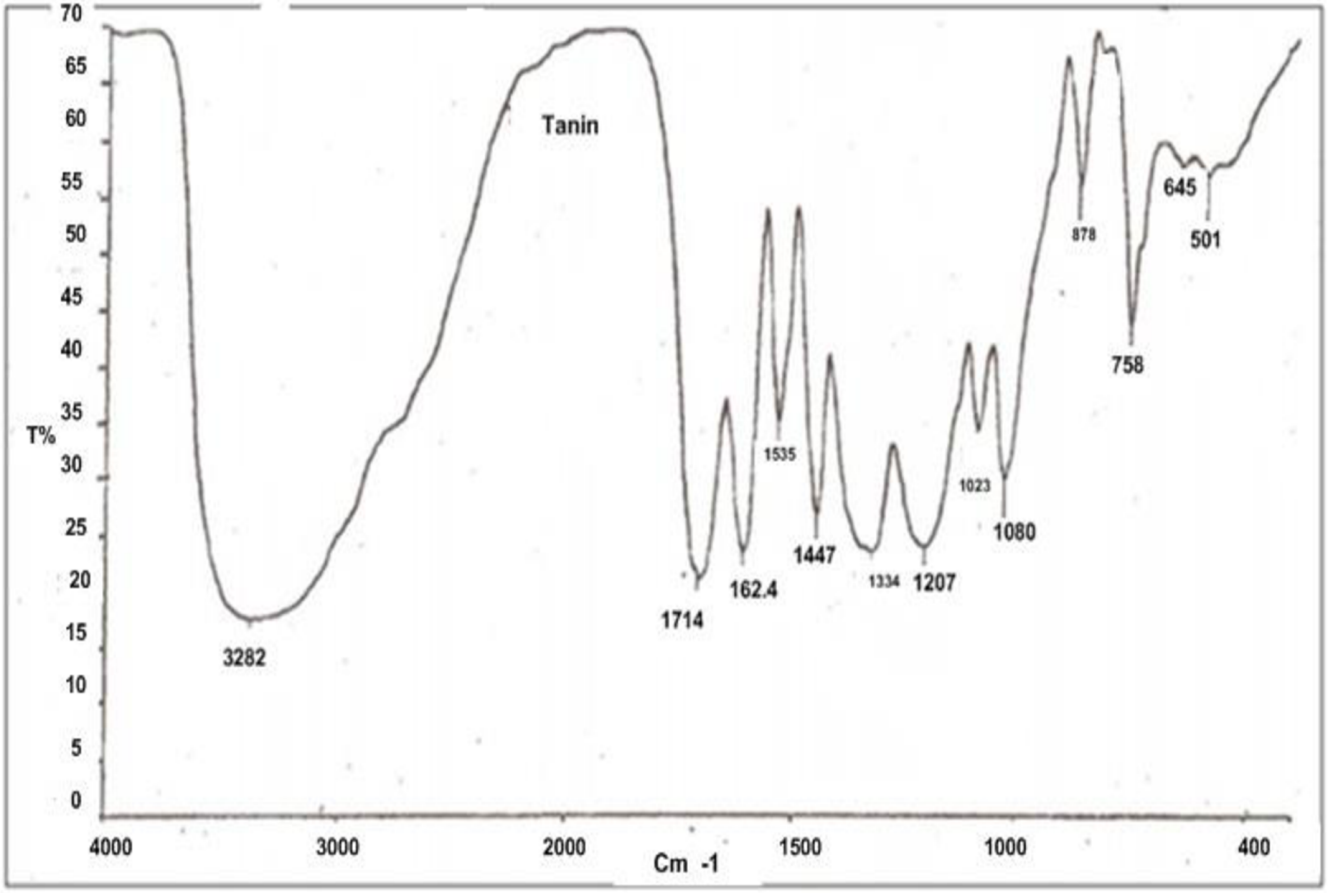
FTIR spectra for BTA-S.

The FTIR result of BTA-S is shown in Fig 4. The maximum absorbance was at 3382 cm^-1^ showing the presence of the hydroxyl group which is in agreement with the work of Rahim et al. [3, 12]. The aromatic compound characteristics were present at peaks between 1714–1030 cm^-1^ while smaller peaks between 870–591 cm^-1^ corresponded to the substituted benzene group [3, 12, 16].

Comparing both results, the trend between RMT and BTA-S is similar. The treated RMT was not pure as the commercial tannin, it might still contain some impurities like pigment and fat which might be the reason for the slight difference in the peaks.

### 3.2 Corrosion rate result and analysis

#### 3.3 Effect of concentration on corrosion rate

**Fig 5:**
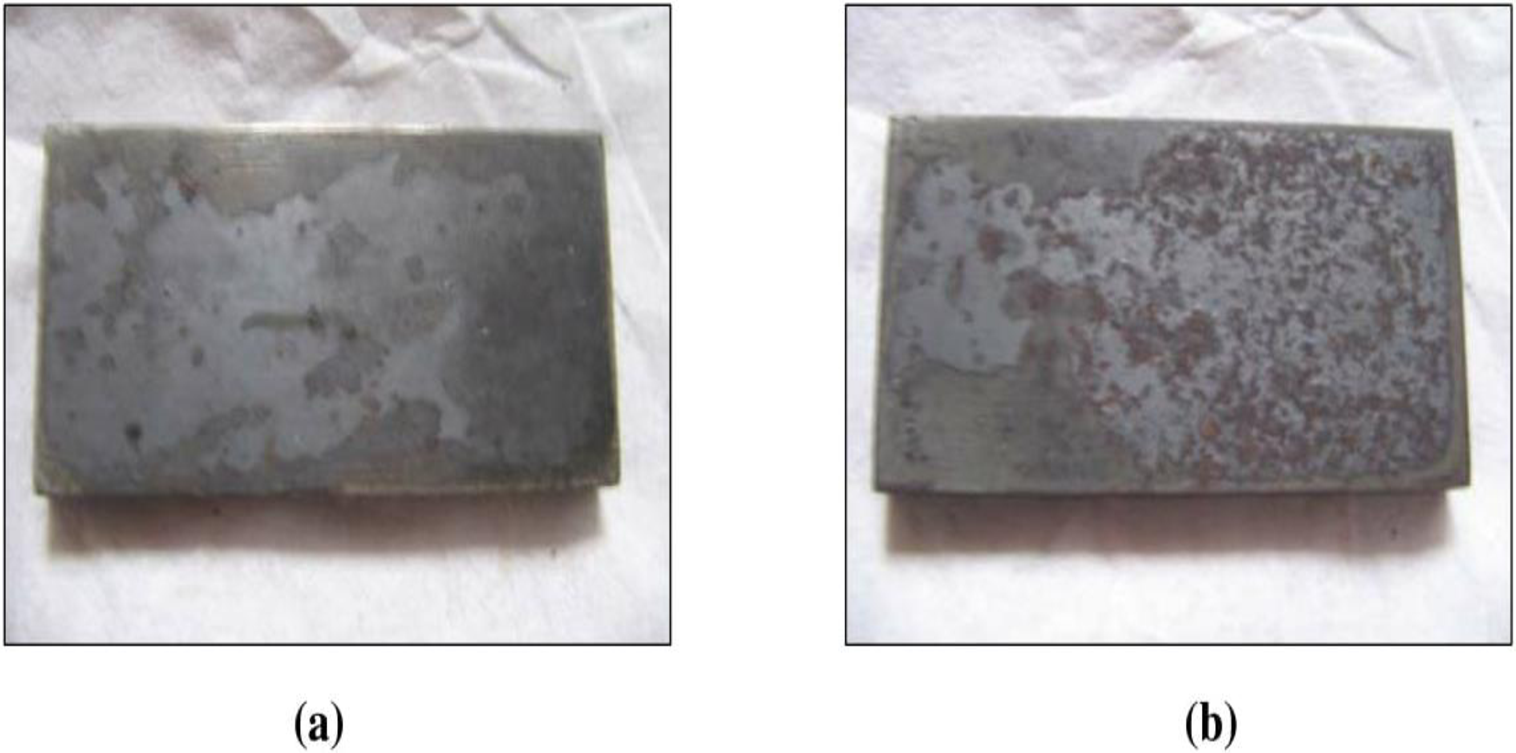
Carbon steel specimen surface after corrosion rate test for 2 weeks period. (a) Without Treated Tannin Presence (b) With Treated Tannin Presence.

**Fig 6:**
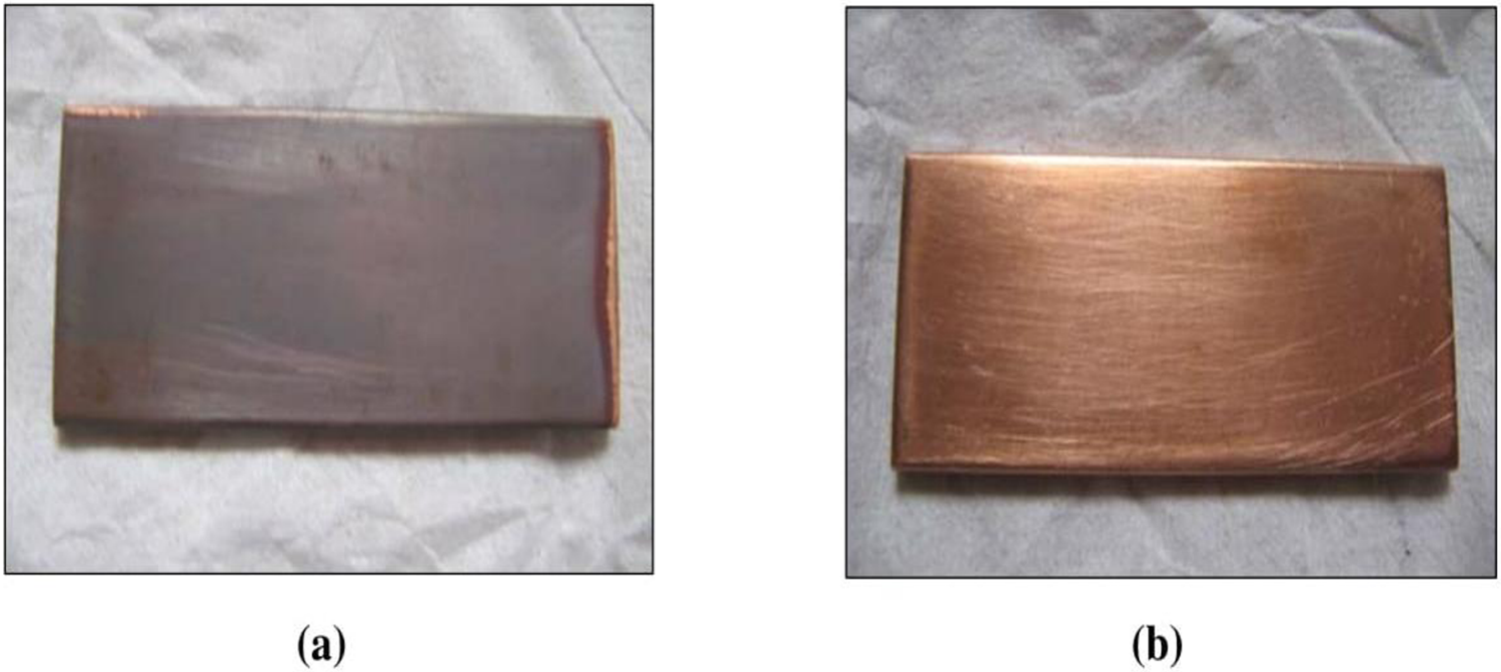
Copper specimen surface after corrosion test for 2 weeks period. (a) Without Treated Tannin Presence (b) With Treated Tannin Presence.

**Fig 7:**
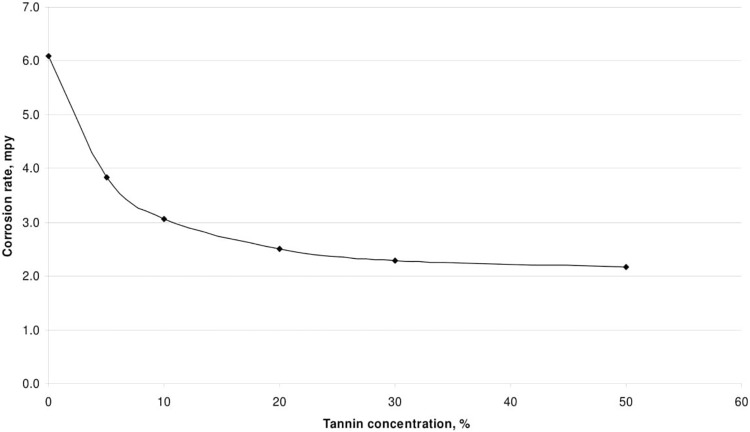
Corrosion rate vs tannin concentration for carbon steel.

**Fig 8:**
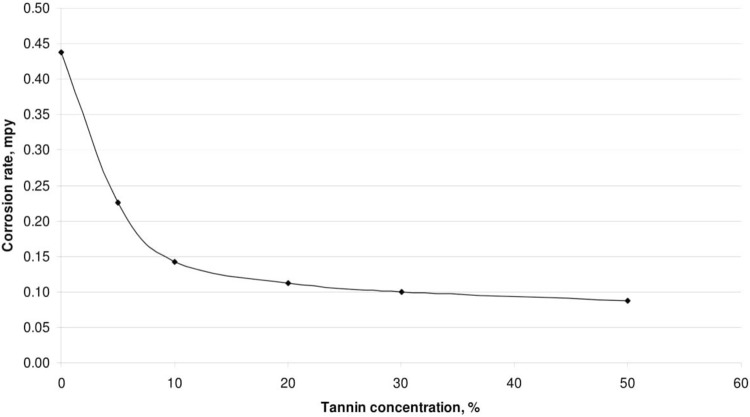
Corrosion rate vs tannin concentration for copper.

The effect of corrosion can be clearly seen at the surface of the specimen used. The corrosion effect was more severe on the surface of the specimen without tannin present in the electrolyte. Figs 5 and 6 show carbon steel and copper specimen surfaces after corrosion test for 2 weeks period. The inhibition capacity of RMT increased with concentration from 5% to 20% after which it became constant for both carbon steel and copper (Figs 7 and 8), similar trend was also observed by Rahim et al. [20]. Therefore, corrosion rate of both metals decreased with increase in concentration, this is because organic compounds containing N, S, O form a co-ordinate type of bond with the metal and the lone pair of electron present in the tannin, this can be enhanced by increasing the effect of the electron by increasing its density at the functional group of the RMT [21, 22, 23]. The mechanism of hydrogen evolution reaction becomes the same as the concentration increases, which leads to more tannin molecules to attached on the surface of the carbon steel and copper, attracting more uniform surface coverage which decreases the rate of hydrogen reduction [3].

The corrosion inhibiting effect of RMT can be attributed to the presence of flavonoid monomers (catechin, epicatechin, epigallocatechin, epicatechin gallate) present in the mangrove plant. These monomers may react with the freshly generated Fe^2+^, ions on the corroding metal surface resulting the formation of organometallic (Fe-Inh) complexes [11, 37, 38, 39].

#### 3.4 Comparison between the weight-loss and electrochemical methods

**Fig 9:**
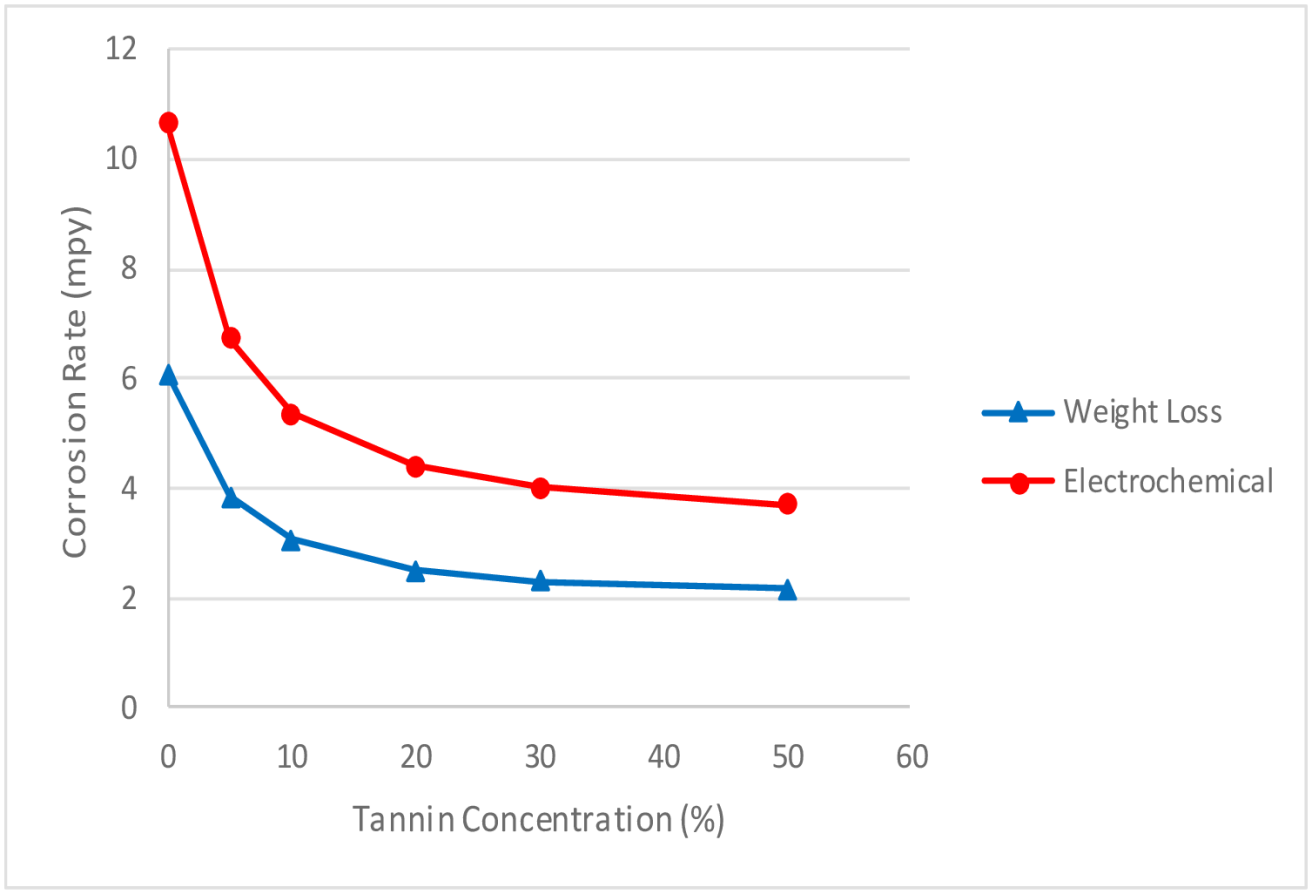
Comparative plot of weight-loss and electrochemical method for carbon steel.

**Fig 10:**
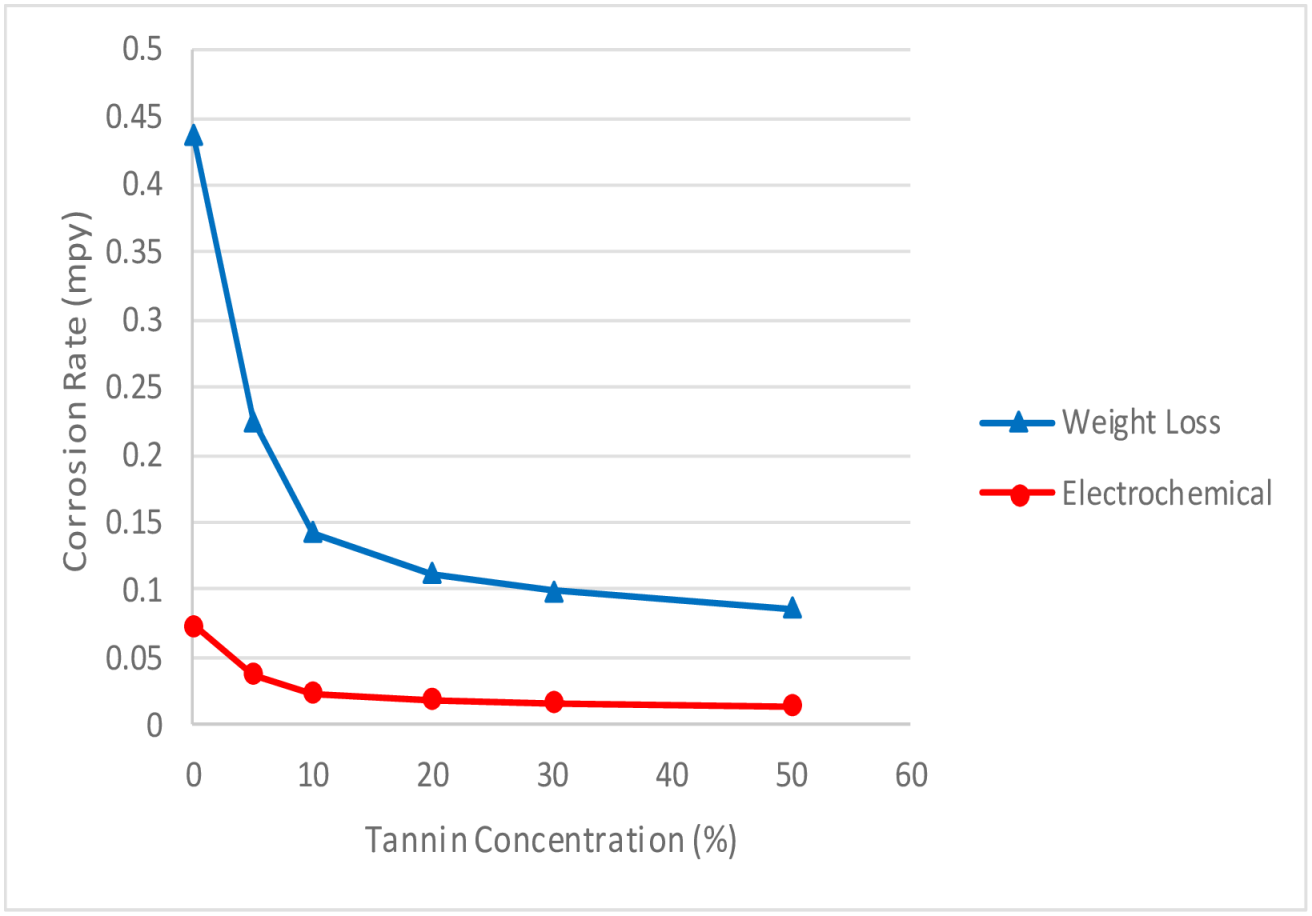
Comparative plot of weight-loss and electrochemical method for copper.

The application of Faradays law is useful for the conversion of corrosion rate data from one system to another. The essence is to compare the results between the different systems analytically. It serves as a quick comparison tool in the absence of experimental results. Figs 9 and 10 show the comparison between the weight-loss and electrochemical method for carbon steel and copper, respectively. It can be observed that for both methods the corrosion rate decreased with increase in concentration. Although, the trends are similar, but the results are far apart. This is because for mild and low alloy steels like carbon, it is assumed that the corrosion rate is uniform. Also, anaerobic activities could be negligible during the 1 year’s immersion [40]. Therefore, the electrochemical activity of the rust layers may be the main reason for the over-estimated electrochemical corrosion rate. The cathodic and anodic corrosion reactions are performed equivalently at the corrosion potential in the weight-loss measurement. Whereas, for the electrochemical measurement, the cathodic and anodic polarisation are carried out to some extent. Which leads to the electrochemical corrosion reactions to be different from those at the corrosion potentials [30]. This could induce some reactions not available at the steady state, leading to different reactions between the electrochemical test and those of the equilibrium state. Resulting to over estimation of the corrosion (Fig 9). The β-FeOOH, which is produced after a long period of immersion reacts with the electrochemical activity in the inner rust layer. Exerting a significant influence on the electrochemical test, even a small polarisation can make the β-FeOOH participate in cathodic reaction, which leads to overestimation of corrosion rate [30]. Also, the rust layer during the early stage of immersion also contain γ-FeOOH and a small quantity of β-FeOOH. The coexistence of these two components can play a major role in accelerating the corrosion rate [41]. The major difference (Fig 9) is that when the weight loss method is performed, the quantity of reduction potential of β-FeOOH is slightly lower than the corrosion potential. This quantity is so small that it those not influence the corrosion reaction. Whereas, in the electrochemical, cathodic polarisation is carried out and this process strengthen the reduction of β-FeOOH which increases the cathodic corrosion rate. As such, the electrochemical result is higher than the weight loss [30].

The rates calculated from these data represents the integral corrosion rates over the examined time period. After applying the Faraday’s law and the apparent valence, the total dissolution (corrosion) current density was determined. The weight loss method has higher corrosion rate than that calculated from the electrochemical (Fig 10). Which is about 6 times higher than the electrochemical corrosion density. The reason for this might be the corrosion rate due to ‘anomalous’ dissolution of copper ions concentration. The finding is consistence with previous studies of Drazic and Popic [42] and Florianovich [43]. They reported that during anodic dissolution more material dissolve than expected from the Faraday’s law with the use of the expected valence of the formed ions. It has been established that alkali metals such as nickel, chromium, titanium, copper, during dissolution passes through a stage of a simple monovalent ion in a form Me^+^. Also, the chemical reaction in which water molecules with metals form metal ions and gaseous hydrogen in a potential independent process. It occurs simultaneously with the electrochemical corrosion process, but the anomalous behaviour is dominantly chemical dissolution. Which is considerably faster than the electrochemical corrosion [44]. Since the electrochemical reaction cannot be followed by electrochemical means, the electrochemical method might give a much smaller corrosion rate than that determined by the weight loss measurement [45]. This implies that the valence used in the Faraday’s law is larger than actually deposited by the metal [44]. Over this observed period, the real corrosion rate was constant and the mechanism by which copper ions was produced did not change the kinetics.

#### 3.5 Effect of concentration on inhibitor efficiency

From Tables 1 and 2 it was observed that, the percentage of inhibition efficiency increased with increase in concentration of the inhibitor. The corrosion-inhibiting efficiency can be attributed to the flavonoid monomers of the tannin. These constituents may react with the freshly generated Fe^2+^ ions on a corroding metal forming organometallic (Fe-Inh) complexes [6]. The resulting Fe-RMT complexes may inhibit or even catalyse further metal dissolution. This is dependent on the complex solubility. The Fe-RMT complexes formed an insoluble surface layer which separated the metal surface from aggressive anions present in the solution [46]. The inhibitive efficiency can also be attributed to the reaction of ferric ion with the polyphenolic fraction of the RMT molecule, resulting in the formation of a highly cross-linked network of ferric tannate moieties [46, 47, 48]. Which protected the metal surface from corrosion. The functional groups are also capable of chelating with the ferric ions, thereby facilitating a strong coordination on the metal surface. Therefore, the integrity of the protective film formed by RMT is dependent on concentration [49].

**Table 1:** Effect of concentration on corrosion rate and inhibitor efficiency of carbon steel specimen at room temperature (26°C).

Treated Tannin Concentration (%)	Mass Loss (g)	Corrosion Rate (mpy)	Inhibitor Efficiency (%)
0	0.158	6.0832	
5	0.0998	3.8424	37
10	0.0795	3.0608	50
20	0.0653	2.5141	59
30	0.0596	2.2947	62
50	0.0562	2.1638	64

**Table 2:** Effect of concentration on corrosion rate and inhibitor efficiency of copper specimen at room temperature (26°C).

Treated Tannin Concentration (%)	Mass Loss (g)	Corrosion Rate (mpy)	Inhibitor Efficiency (%)
0	0.0116	0.4373	
5	0.006	0.2256	48
10	0.0038	0.1424	67
20	0.003	0.1122	74
30	0.0026	0.0996	77
50	0.0023	0.087	80

#### 3.6 Effect of temperature on corrosion rate

**Fig 11:**
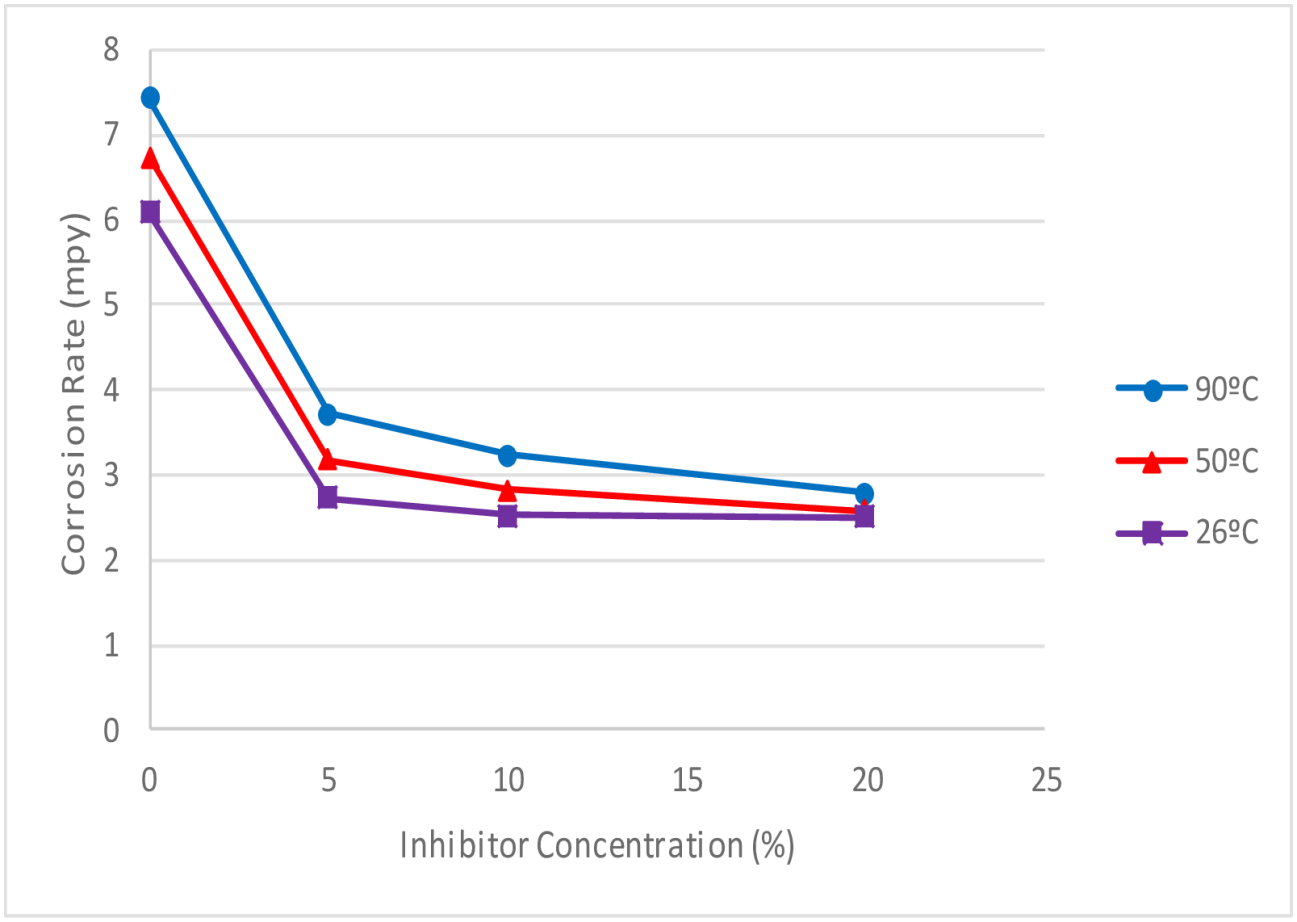
Corrosion rate vs Optimum Tannin Concentration of carbon steel at 26°C, 50°C and 90°C.

**Fig 12:**
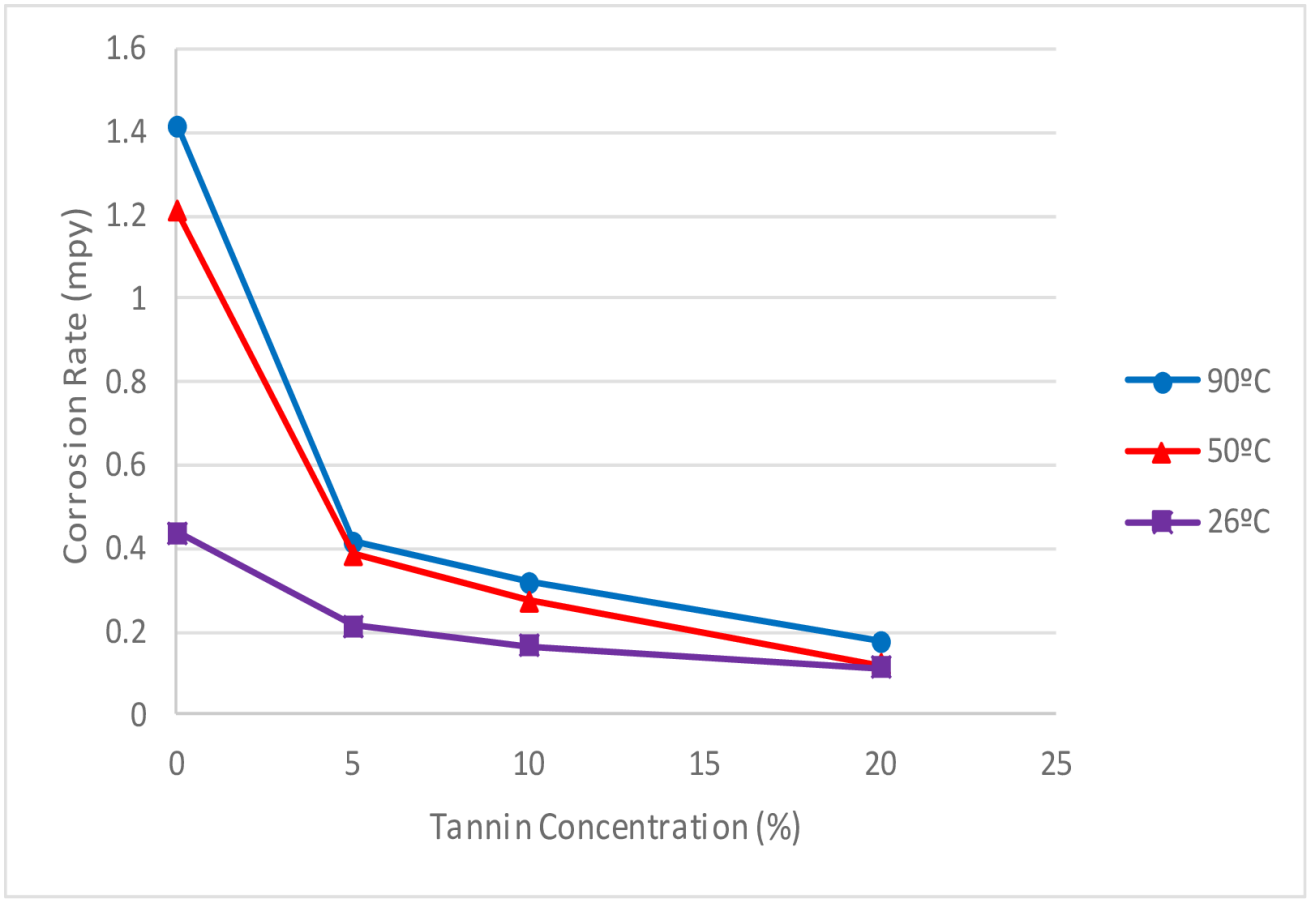
Corrosion rate vs Optimum Tannin Concentration of copper at 26°C, 50°C and 90°C.

The corrosion rate for carbon steel at 26°, 50° and 90°C is shown in Fig 11, while that of copper is shown in Fig 12. The tannin concentration used in this test is the best concentration of 20% (w/v). From the graph, the corrosion rate increased with increase in temperature for both metals, this confirms previous studies [6, 50]. However, the corrosion rate was less severe when tannin was present in the electrolyte, which can be attributed to the protective film formed by the RMT. Therefore, RMT is good potential as a corrosion inhibitor.

#### 3.7 Effect of temperature on inhibition efficiency

Tables 3 and 4 summarise the results of the effect of temperature on corrosion rate and inhibition efficiency with and without treated RMT for carbon steel and copper respectively. From the Tables, inhibition efficiency decreases with increase in temperature for both metal studied, this is in agreement with previous studies [6, 50]. The possible reason for this is the desorption of some of the inhibitor from the metal surface at higher temperature, which indicates that the inhibitor was physically attached to the metal surface [48].

**Table 3:** Effect of temperature on corrosion rate and inhibitor efficiency of carbon steel.

Concentration (%)	Mass Loss (g)	Column1	Column2	Corrosion Rate (mpy)	Column3	Column4	Inhibition Efficiency (%)	Column5	Column6
	90°C	50°C	26°C	90°C	50°C	26°C	90°	50°C	26°C
0	0.1934	0.175	0.158	7.4448	6.7377	6.0832			
5	0.0964	0.0826	0.0707	3.7142	3.1822	2.7241	39	48	55
10	0.08402	0.0737	0.0657	3.2351	2.831	2.531	47	53	58
20	0.0725	0.0669	0.0653	2.79	2.5757	2.5141	54	58	59

**Table 4:** Effect of temperature on corrosion rate and inhibition efficiency of copper specimen.

Concentration (%)	Mass Loss (g)	Column1	Column2	Corrosion Rate (mpy)	Column3	Column4	Inhibition Efficiency (%)	Column5	Column6
	90°C	50°C	26°C	90°C	50°C	26°C	90°	50°C	26°C
0	0.0375	0.032	0.0116	1.4177	1.2098	0.4373			
5	0.011	0.0102	0.0056	0.4159	0.3867	0.2117	5	12	52
10	0.0084	0.0072	0.0044	0.3176	0.2721	0.1663	28	39	62
20	0.0047	0.0032	0.003	0.1777	0.1209	0.1134	59	72	74

#### 3.8 Comparative analysis of RMT with commercial corrosion inhibitor BTA-S

**Fig 13:**
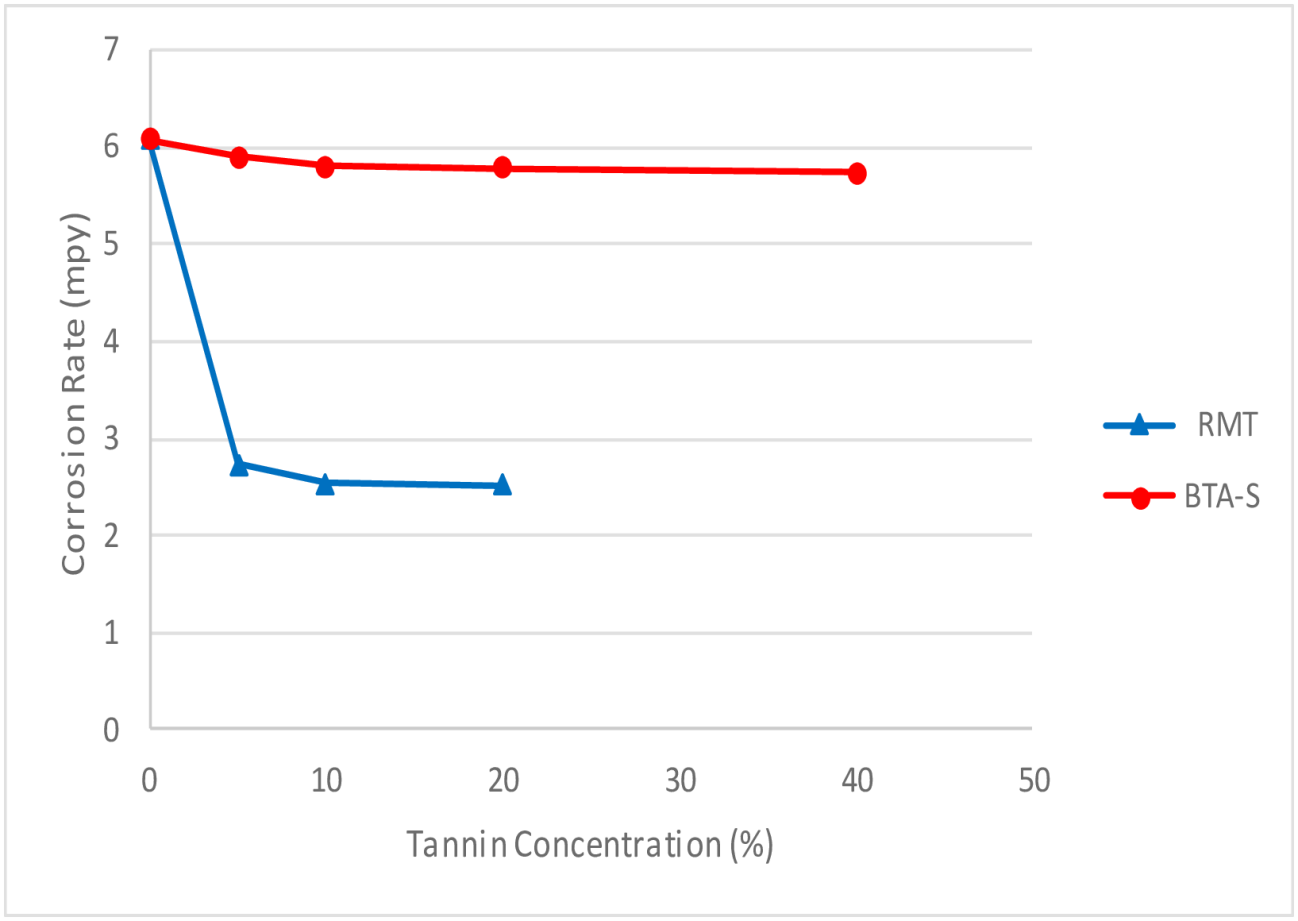
Corrosion rate vs concentration of tannin and BTA-S for carbon steel.

A comparison in terms of inhibition performance was made between the treated RMT and BTA-S. Commercially, BTA-S is effective in reducing the corrosion rate on copper plate or copper alloys. Fig 13 show the corrosion rate between RMT and BTA-S for carbon steel. From the plot, the results between the two inhibitors were far apart, even though the concentration of BTA-S used in the test was higher than that of treated RMT. For the inhibitor efficiency, RMT gave 59% efficiency to reduce corrosion rate on carbon steel while BTA-S gave only 6% efficiency. Therefore, BTA-S is not suitable to be used as a corrosion inhibitor to reduce corrosion rate for carbon steel.

**Fig 14:**
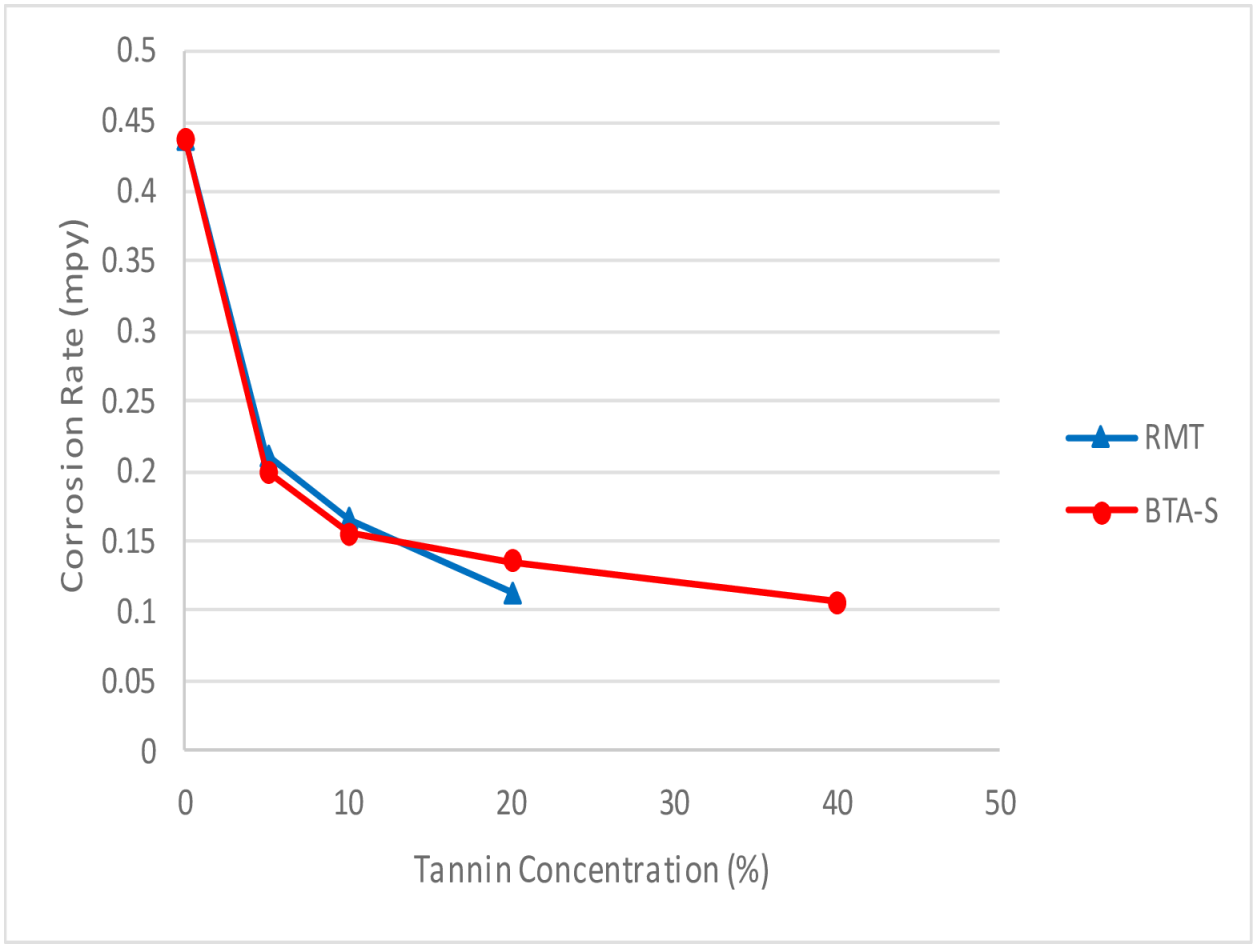
Corrosion rate vs concentration of tannin & BTA-S for copper.

The corrosion rate between RMT and BTA-S for copper is shown in Fig 14. From the graph, the performance between treated RMT and BTA-S was almost similar. For the inhibition efficiency, treated RMT gave a 74% efficiency to reduce corrosion rate on copper while BTA-S gave 76% efficiency. Therefore, RMT reduced the corrosion rate on copper better than BTA-S. This is because the difference between both inhibitors was not much, even when the concentration of BTA-S used was 20% (w/v) higher than that of RMT. Details of the experimental results are shown in Tables 5 and 6 below.

**Table 5:** Corrosion rate and inhibitor efficiency of treated tannin and BTA-S for carbon steel.

Concentration (%)	Mass Loss (g)	Column1	Corrosion Rate (mpy)	Column2	Inhibition Efficiency (%)	Column3
	RMT	BTA-S	RMT	BTA-S	RMT	BTA-S
0	0.158	0.158	6.0832	6.0832		
5	0.0707	0.1552	2.7241	5.9741	32	2
10	0.0657	0.1523	2.531	5.8635	46	4
20	0.0653	0.1505	2.5141	5.7962	59	5
40		0.1489		5.7328		6

**Table 6:** Corrosion rate and inhibitor efficiency of treated tannin and BTA-S for copper.

Concentration (%)	Mass Loss (g)	Column1	Corrosion Rate (mpy)	Column2	Inhibition Efficiency (%)	Column3
	RMT	BTA-S	RMT	BTA-S	RMT	BTA-S
0	0.0116	0.0116	0.4373	0.4373		
5	0.0056	0.0053	0.2117	0.2003	52	54
10	0.0044	0.0041	0.1663	0.155	62	65
20	0.003	0.0036	0.1134	0.136	74	69
40		0.0028		0.1059		76

**Fig 15:**
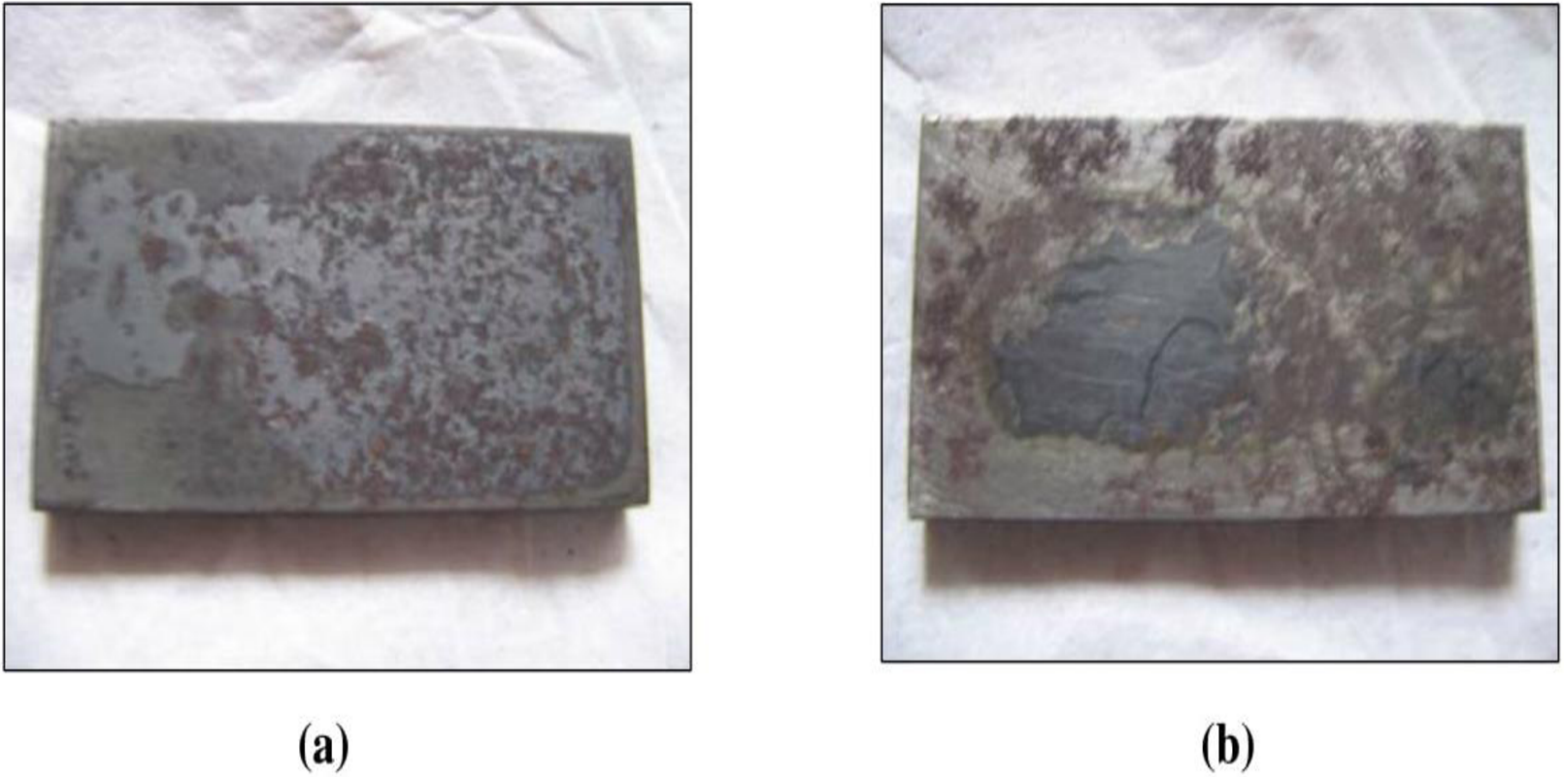
Carbon steel specimen surface after corrosion test for 2 weeks period at room temperature. (a) With Treated RMT, (b) With BTA.

**Fig 16:**
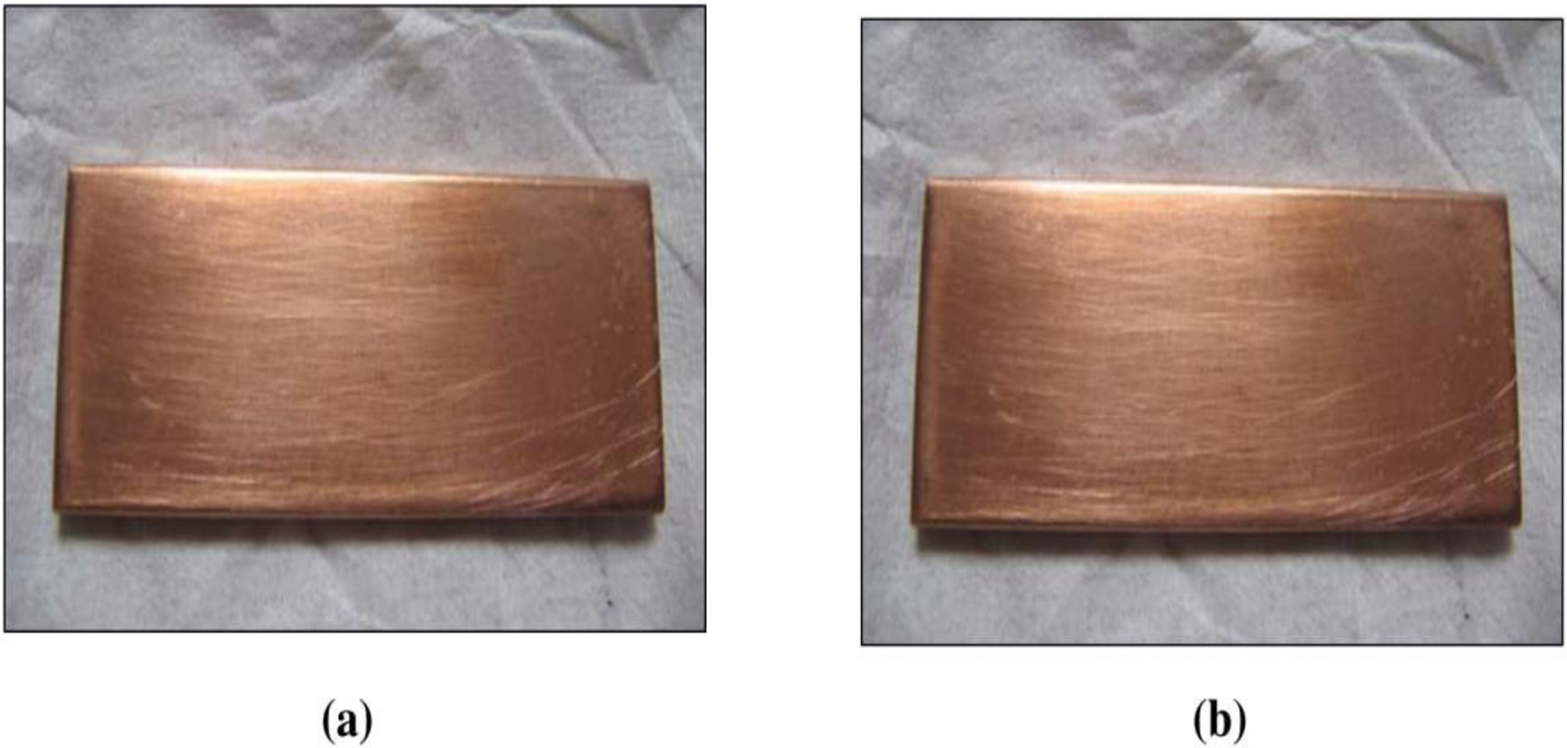
Copper specimen surface after corrosion test for 2 weeks period at room temperature. (a) With Treated RMT, (b) With BTA-S.

Figs 15 and 16 display the image of carbon steel and copper specimen surface after corrosion test for 2 weeks period using RMT and BTA-S at room temperature.

## Conclusions

Based on the results of this study, the following conclusion were made. The FTIR result of RMT was similar to that of commercial inhibitor BTA-S. The best concentration for treated RMT at room temperature was 20% (w/v). Increase in concentration increased the inhibition capacity of RMT until it reached a plateau where further increase has no effect. The major difference between the weight loss measurement and the electrochemical method on carbon steel is that when the weight loss method is performed, the quantity of reduction potential of β-FeOOH is slightly lower than the corrosion potential. This quantity is so small that it those not influence the corrosion reaction. Whereas, in the electrochemical, cathodic polarisation is carried out and this process strengthen the reduction of β-FeOOH which increases the cathodic corrosion rate. As such, the electrochemical result is higher than the weight loss. But with copper, since the electrochemical reaction cannot be followed by electrochemical means, the electrochemical method might give a much smaller corrosion rate than that determined by the weight loss measurement. Corrosion rate increased with increase in temperature for both metals, but the corrosion rate was less severe with RMT while, the inhibitor efficiency decreased with increase in temperature for both metals. Commercial inhibitor BTA-S can be used as a corrosion inhibiting agent for copper and not suitable for carbon steel. Based on the experimental results, RMT is suitable as a corrosion inhibiting agent for both carbon steel and copper. Therefore, its use as a commercial inhibitor is proposed.
